# Non-visual opsins and their role in circadian photoentrainment

**DOI:** 10.1186/s40662-025-00470-0

**Published:** 2026-01-03

**Authors:** Ethan D. Buhr, Russell N. Van Gelder

**Affiliations:** 1 Department of Ophthalmology, Campus Box 358058, University of Washington School of Medicine, 750 Republican St, Seattle, WA 98109 USA; 2 Department of Neurobiology & Biophysics and Laboratory Medicine & Pathology, University of Washington School of Medicine, Seattle, WA USA; 3 Roger and Angie Karalis Johnson Retina Center, University of Washington School of Medicine, Seattle, WA USA

**Keywords:** Circadian rhythm, Biological clocks, Photoentrainment, Entrainment, Opsin, Opn4, Opn5, Opn3, Non-visual

## Abstract

Photoreception is common in animals without a visual system. In animals with visual systems, it is sometimes presumed that the same photoreceptors and pathways will accommodate both visual and non-visual light detection. However, mounting evidence reveals that most animals exhibit broad extra-visual photoreceptive functions that are wholly independent of the visual system. One of these functions is the synchronization of the circadian clock to light–dark signals, or photoentrainment. In mammals, behavioral photoentrainment is achieved exclusively through visual and non-visual opsin proteins within the retina, and molecular photoentrainment of individual cells occurs using non-visual opsins in some peripheral tissues. This is in contrast to insects and fish where nearly all peripheral organs are directly photoentrainable. This review will summarize the family of opsins in mammals and focus on the role of non-visual opsins in circadian photoreception. Particular emphasis will be given to photoentrainment in other vertebrates in order to compare and contrast the use of the wide range of non-visual opsins in circadian photoentrainment throughout the animal kingdom.

## Background

In the late nineteenth century, scientists began documenting evidence of extra-visual photoreception in the animal kingdom. In 1847, Édouard Brown-Séquard observed that the pupil of an eel’s eye continued to constrict in response to light after the eye had been isolated from the animal [1, 2]. Subsequently in 1890, Sir Edward Bagnall Poulton demonstrated that small tortoiseshell caterpillars changed their skin color to match their surroundings even when blinded or when unable to see their surroundings with their eyes [3]. These early observations provided evidence that animals with visual systems exhibited light-driven functions independently from the common pathways of image-forming vision. One of these extra-visual phenomena is the synchronization of near-24-h behavioral rhythms, or circadian rhythms, to the rotation of the earth. Nocturnal (active at night), diurnal (active in day), and crepuscular (active at sunrise and sunset) animals all use light cycles to maintain a consistent phase relationship to their environment. In fact, the phase of the molecular oscillations the central brain clocks of nocturnal, diurnal, and crepuscular mammals is unified relative to sunrise and sunset, not to the behavior of the individual species [4]. Light exposure near sunrise or sunset is critical for animals to maintain their behavioral niche in an elegant light-response system shared by nearly all animals [5]. Briefly, light exposure near sunrise nudges the clock forward, and exposure near sunset nudges it backward [6–8]. This “phase response curve” is sufficient to push the light-sensitive circadian clock to its appropriate phase in nearly all organisms regardless of whether their main activity bout occurs at day or night [9]. The environment we have created for ourselves as humans includes abundant electric light after sunset, travel across many time zones in a single day, and social and professional pressures that often go against our internal sleep drive. These activities confuse our systems of circadian photoreception in ways that require constant adaptation and may cause misalignment. This review will focus on the mechanisms animals use to achieve extra-visual circadian synchrony, with special focus on non-visual opsins.

## Main text

### Non-visual opsins

**Fig. 1:**
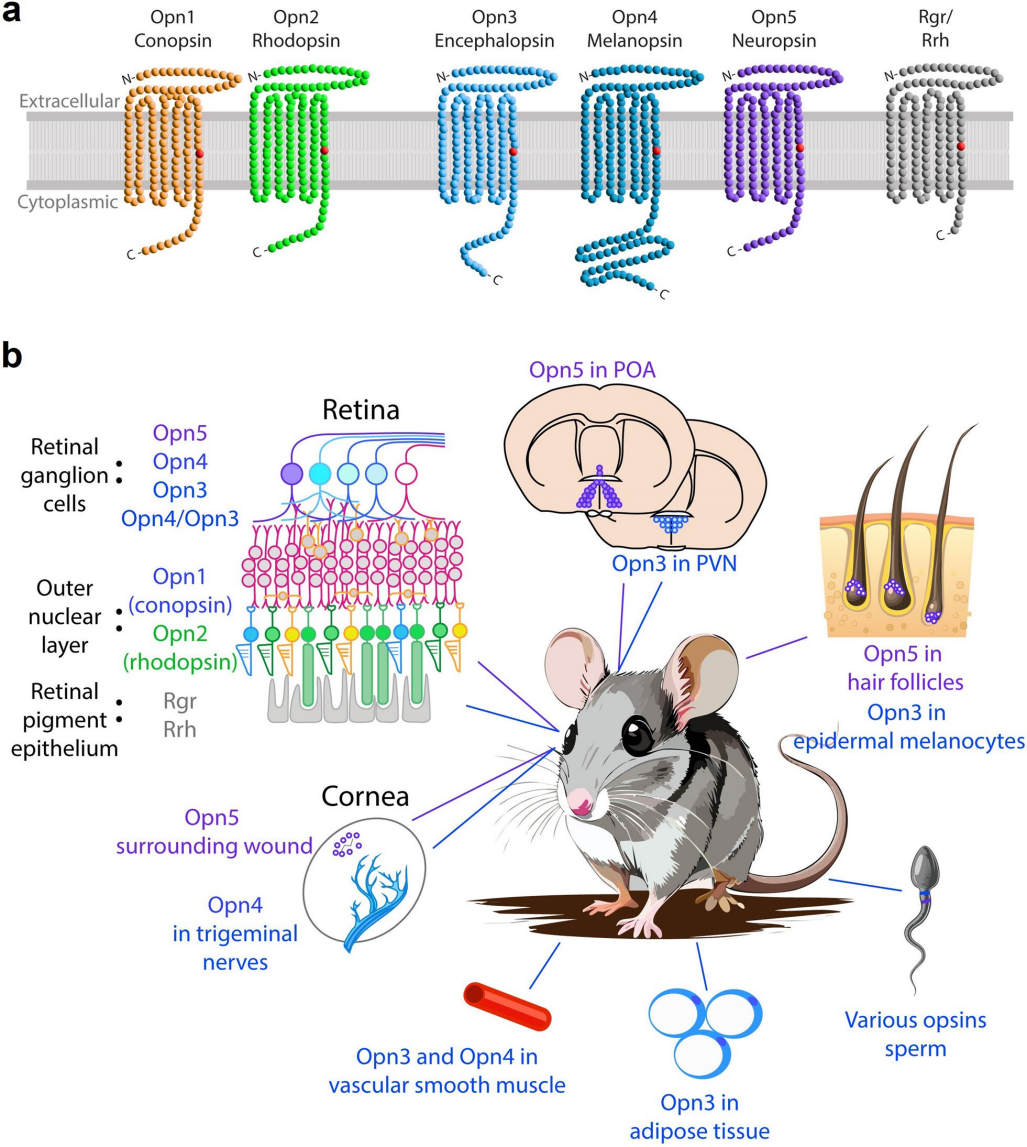
The opsin repertoire of mammals. **a** Most mammals have two classes of visual opsins (conopsin and rhodopsin), three non-visual opsins (encephalopsin, melanopsin, and neuropsin), and opsins which act as photoisomerases and aid in chromophore recycling (Rgr and Rrh). Among the visual opsins, conopsins are further subdivided based on their wavelength sensitivity: “*OPN1*sw” for short wavelength, “*OPN1*mw” for middle wavelength, and “*OPN1*lw” for long wavelength (observed only in trichromatic animals such as Old World primates including humans and some New World primates such as howler monkeys). All are seven transmembrane receptors with their amino terminus (N-) on the extracellular side and their carboxyl terminus (-C) on the intracellular side of the cell membrane. Each contains a lysine residue in the seventh transmembrane domain (shown as a red point). **b** Locations of visual and non-visual opsins in mice and humans for which some function has been suggested. POA, preoptic area; PVN, paraventricular nucleus

Opsins are proteins of the G-protein coupled receptor (GPCR) family, which are seven transmembrane proteins that activate a heterotrimeric G protein when excited by the GPCR’s preferred ligand. In the case of opsins, the preferred ligand is a light-sensitive vitamin A isoform, retinaldehyde, which covalently binds to the protein’s seventh transmembrane region (Fig. 1a). It is important to briefly review the basic molecular mechanism of mammalian (and most vertebrate) vision to draw distinctions and similarities with extra-visual mechanisms. For vision, cones and rods express opsin 1 (OPN1) or opsin 2 (OPN2), respectively. A retinaldehyde molecule in the 11-*cis* isomeric form binds to OPN1 or OPN2 to form a photopigment in the inactive state, primed to signal a photon catch. When light converts the retinaldehyde molecule from 11-*cis* to an all-*trans* state, the protein enters an active state (also called “Meta II”) in which the opsin can activate its preferred G-protein, which for visual opsins is transducin, or G_t_ [10]. To simplify decades of research on the biochemistry of rod- and cone-based vision: the activation of G_t_ leads to a decrease in intracellular cyclic guanosine monophosphate (cGMP), leading to the closing of cyclic nucleotide-gated cation channels, the cell’s membrane voltage hyperpolarizes, and the light event is signaled synaptically through the retina. During this process, the all-*trans* retinaldehyde is released from the opsin and is shuttled either to the retinal pigment epithelium (RPE) or to Müller glia cells to be recycled [11]. Opsins are typically categorized based on their amino acid structure, which dictates their associated G-alpha protein and their peak spectral sensitivity, or “λ-max”. The λ-max is determined by the push and pull of charges on amino acid residues near the bound retinaldehyde moiety [12].

OPN1 and OPN2 belong to a class of opsins called ciliary opsins due to the type of ciliated cells in which they are typically expressed in vertebrates, whereas rhabdomeric opsins were identified in the rhabdomeres of invertebrate visual systems [13]. Despite the names, vertebrates express opsins of both classes as well as opsins of the neuropsin and G_o_/photoisomerase family [10, 14]. The rhabdomeric and neuropsin opsins have recently been found to play critical roles in extra-visual photoreception in mammals and other vertebrates. This review will focus primarily on the six to seven opsins present in the mammalian genome with special emphasis on the three verified mammalian non-visual opsins: Opn3, Opn4, and Opn5 (Fig. 1b). Many more opsins can be found in amphibians (about 14 opsins) [15], birds (about 13) [16], and fish (10–42, varying greatly by species) [17–20], and discussion of each opsin is beyond the scope of this review.

#### Opn3

OPN3 was identified by Blackshaw and Snyder in murine brain and gonads [21]. They used nucleotide sequence of an amphibian non-visual opsin to screen for novel opsin RNA sequences among mammalian expressed sequence tags and then cloned the complementary DNA (cDNA) of a relevant mammalian match. They then used an RNA-probe to search for this transcript in mouse tissues. Because they observed the highest abundance of transcript of this novel murine opsin in the brain, they named it “encephalopsin” [21]. Using a separate RNA-probe, another group discovered *Opn3* expression in a wider range of tissues including brain, retina, gonads, and some internal organs and dubbed the opsin “panopsin” [22, 23]. Sequence identity places OPN3 in a class of opsins closely related to Teleost Multiple Tissue (TMT) opsins originally identified in fish [24]. As its name suggests, TMT is found in organs throughout a fish’s body, with its broad extraocular expression marking another similarity to OPN3 in addition to amino acid sequence [25]. In mammals, the expression of *Opn3* in adipose tissue and skin has caused fresh interest in this opsin. In white adipose tissue, *Opn3*-dependent lipolysis and thermal regulation link this opsin to systemic metabolic regulation in mice [26]. Its expression in the paraventricular nucleus of the hypothalamus is associated with regulation of food consumption, furthering its role in energy homeostasis [27]. In melanocytes from human skin, *Opn3* acts as a negative regulator of melanin production [28].

Much is unknown about the light-signaling properties of mammalian OPN3, and much is inferred from its phylogenetic relationship to TMT/OPN3 opsins in other species. For example, the peak spectral sensitivities of mosquito, zebrafish, and pufferfish OPN3/TMT opsins are about 470 nm in heterologous expression [29, 30]. The spectral sensitivity of mammalian OPN3 is often reported to be in this same range, although the difficulty in obtaining light-responses from cells heterologously expressing mouse or human OPN3 leaves this open for further analyses [30]. The preferred G-alpha protein of OPN3 is also unclear, but is likely G_i/o_. In human skin, OPN3 activity is associated with G_i/o_ signaling, although this may be due to an association with the G_i/o_-activating melanocortin receptor, MC1R [28]. Similarly, OPN3 in the hypothalamus is associated with G_i/o_ signaling, but in these cells they are associated with the melanocortin 4 receptor (MC4R) [27]. However, MC4R typically activates G_s_ signaling, strengthening the likelihood that the G_i/o_ signal in Opn3-PVN neurons may be Opn3-mediated. In heterologous systems, fish TMT opsins and mosquito Opn3 activate G_i/o_ protein pathways [29]. While TMT opsins and OPN3 cluster together phylogenetically and appear to share associated G-protein pathways, these two opsin types may not be completely interchangeable [25, 29, 30]. For example, zebrafish have retained and express both [24]. Most mammals express only OPN3, but monotremes express only TMT2-opsin [31]. Marsupials stand out as the only mammals that express both OPN3 and teleost-like TMT2 [24, 31]. The evolutionary advantage for animals expressing one or the other or both variants is unknown. The expression of TMT/OPN3 opsins throughout the body of both fish and mammals and their physical association with melanocortin receptors suggest interesting, novel physiology.

#### Opn4

OPN4 is the most extensively studied of the non-visual opsins in mammals. It was first discovered in 1998 in frog (*Xenopus laevis*) melanophores by Ignacio Provencio, Mark Rollag, and colleagues [32]. Frog melanophores can be maintained in vitro, and they respond directly to light by redistributing the dark pigment melanin from around the nucleus to the extended pseudopods [33, 34]. There was early evidence that this action was opsin-based in that this phenomenon required the presence of retinaldehyde [35]. Provencio et al. analyzed *Xenopus* melanophores for immunoreactivity using antibodies for bovine rhodopsin and conducted a search for novel opsin transcripts in cDNA screens to demonstrate the presence of an opsin [32]. They were able to deduce the protein sequence and predicted structure of this novel opsin, which they named “melanopsin” after the cells in which it was discovered. Interestingly, the sequence of melanopsin placed it into the rhabdomeric clade of opsins, meaning it was more similar to typical invertebrate opsins than to the ciliary opsins associated with vertebrate vision [14, 36]. A mammalian version of OPN4 was then identified in a subset of retinal ganglion cells (RGCs) of mammals including rodents [37, 38], non-human primates [39], and humans [40]. Prior to the discovery of melanopsin, the action spectrum of light-mediated melatonin suppression in humans suggested sensitivity in a wavelength range that did not quite match with rod or cone photoreceptors [41, 42]. The spectral peak of sensitivity of mammalian OPN4 is about 480 nm (sky blue in human vision) which, coincidentally, matched the predicted wavelength sensitivity of the putative photoreceptor [43, 44]. The presence of intrinsically photo-sensitive retinal ganglion cells (ipRGC) in the mammalian eye with the correct spectral tuning for melatonin photo-responses was a harbinger of a whole new branch of circadian and sleep research. The function of these cells will be discussed in more detail in the section on mammalian circadian photoentrainment.

Unlike OPN3, mammalian OPN4 generates strong photo-responses when expressed in heterologous systems. Its expression in HEK293 cells, Neuro2A cells, and frog oocytes all pointed to Gq as the preferred G-alpha protein of melanopsin [43–45]. These cell-culture experiments showed that the activation of OPN4 caused an increase in cytoplasmic Ca^2+^ and the activation of a TRPC3 channel [43, 44]. This is similar to the TRP-mediated light responses in invertebrate rhabdomeric opsins [10]. Identifying a single TRPC channel necessary for the response in native ipRGCs has been difficult, potentially due to redundancy and compensation among multiple channels [46, 47]. The removal of TRPC6 and 7 greatly reduced light responses in the M1 class of Opn4-ipRGCs but left pupillary light responses unchanged [48]. The activation cascade appears to not be identical between different subclasses of Opn4-ipRGCs, with some activating T-type Ca^2+^ channels in addition to TRPC [49]. Similar to ciliary opsins, the apoprotein (or protein without chromophore) of OPN4 binds only to *cis* and not *trans* retinaldehyde [45, 50]. However, melanopsin binds tightly to the chromophore through many light events, suggesting a bistable photocycle [50]. Briefly, this means that rather than the chromophore being recycled from *trans* back to *cis* by an accessory cell as in rod and cone photo-transduction, the *trans* (activated) form is isomerized back to the *cis* (inactive) form by another, usually longer, wavelength of light [10]. This allows the opsin to maintain binding with a single retinaldehyde molecule for long periods of time. There is even some suggestion of a tristable mechanism of melanopsin signaling, in which there are two inactive states and one active, allowing integration of a broader wavelength range [13, 51, 52]. The amino acid sequence of OPN4’s long cytoplasmic tail also allows it to avoid rapid deactivation during prolonged light exposure [53]. These features allow integration of light signals over long time spans, as OPN4 expressing ipRGCs can signal the presence of blue light continuously over many hours [54]. OPN4 has also been found in the mammalian trigeminal nerve [55, 56], blood vessels [57, 58], and human skin [59].

#### Opn5

OPN5 is present in fish [60], birds [61, 62], amphibians [60], and all branches of mammals [31]. Interestingly, OPN5 shows the slowest mutation rate (highest level of conservation) among all opsin classes [31]. The laboratory of Robert Lucas identified *Opn5* in 2003 by querying the human genome for novel opsin sequence, and then its transcript was localized to the brain, eye and testes of mouse and human tissue [63]. Because of its presence in human and mouse brain tissue, they gave Opn5 the name “neuropsin”. Later studies localized *Opn5* expression in the mammalian hair follicles in skin [64, 65], RGCs [60, 64, 66], preoptic area of the hypothalamus [60, 67, 68], epithelial cells of wounded corneas [69, 70], and sperm [71]. While this is a less extensive repertoire than OPN3, OPN5 is expressed in multiple tissues, which are typically near the body’s surface (the preoptic nucleus is the notable exception).

*Opn5* can be successfully expressed in heterologous cell lines and render the cells photosensitive. The spectral sensitivity of OPN5 is much shorter in wavelength than OPN4 or TMT/OPN3. Absorbance spectra of fish, chicken, frog, human and mouse OPN5 peak at about 380 nm [60, 61, 64]. This absorbance peak is corroborated by functional activation studies in vivo [65–67]. Human OPN5 expressed in HEK293, Neuro2A, or rat RGCs caused an increase in internal Ca^2+^ and a phosphorylation of extracellular signal-related kinase (ERK) proteins in the mitogen-activated protein kinase (MAPK) pathway [72, 73]. Independent studies converged on G_i_ as the G-alpha pathway associated with OPN5 of multiple species in cell culture [60, 61, 64, 74, 75]. However, recent studies have shown that the calcium response in OPN5 expressing cells is due to G_q/14_ pathways [76, 77]. In mouse hypothalamus OPN5-expressing neurons responded to light with cAMP changes, suggesting G_i/s_ involvement [67]. Divergent results for the associated G-protein could result from tissue specificity and promiscuity of OPN5 for G-alpha proteins.

It is also unclear if OPN5 is a bistable opsin. Similar to OPN4-expressing frog melanophores, OPN5-expressing mouse skin explants require exogenous chromophore for light responses, specifically the *cis* form [65]. In initial tests of OPN5 absorbance, the opsin was able to shift back and forth from a short- and long-wavelength exposure [61]. However, Shichida and colleagues performed a detailed study of OPN5 from various vertebrates and found that mammals alone lost the bistable nature of OPN5 chromophore binding due to a single amino acid mutation [60]. This perhaps explains the need for exogenous chromophore in explanted tissue [65]. Unlike the retina with its supply of retinoids from the retinal pigment epithelium, the source of *cis* retinaldehyde for OPN3, OPN4, OPN5, and other non-visual opsins in extraocular tissues remains unclear in fish, birds, amphibians, and mammals.

Within the mammalian RGC layer, OPN5 is expressed in a small subset of 27 different ganglion cell types [78]. Of these, F-mini ON and HD2 ganglion cells make up about 30% of all OPN5-containing RGCs [78]. When compiling results from an Opn5-Cre lineage marking system, two *Opn5-lacZ* knock-in mouse lines [66], and RNAscope transcript labeling, OPN5 ganglion cells are present at about 500 cells/mm^2^ in the mouse retina [78]. This compares to about 133 cells/mm^2^ of OPN4-containing ganglion cells using a similar Cre-based reporter [79], and about 200 cells/mm^2^ expressing OPN3 as measured by a transgenic green fluorescent protein (GFP) reporter [80]. Caution is always warranted when comparing between measurement modalities. OPN5 is not co-expressed in the same ganglion cells as OPN4 or OPN3. However, OPN3 and OPN4 share expression in about 24% of OPN3-expressing RGCs [80]. The central projections of OPN4 and OPN5 RGCs are also very distinct. OPN4 cells (which are classified into about six categories named M1–M6 [49]) heavily innervate the suprachiasmatic nucleus (SCN) of the hypothalamus, the olivary pretectal nucleus (OPN), the intergeniculate leaflet (IGL), and the perihabenular nucleus (PHb), among other areas [81]. OPN5-containing RGCs innervate central brain regions primarily related to vision and accessory optic regions, such as the ventral and dorsal lateral geniculate nuclei (LGN), superior colliculus, OPN, and medial and dorsal terminal nuclei [78]. The OPN is one of the few brain structures that receive both OPN4 and OPN5 projections. While the role of central projections of OPN4-containing RGCs has been extensively studied and will be discussed below, little is known about the role OPN5 plays in retina-to-brain signaling. From our research and that of others, OPN5 signaling has stronger effects on cells in the local tissue niche than on distant axonal targets. *Opn3*-based reporters reveal neural projections throughout the mouse brain, but because expression of *Opn3* is also present in several brain nuclei, it is difficult to discern which labeled fibers are specifically projections of OPN3-containing RGCs [82, 83]. A retina-specific labeling system or careful tract-tracing will be required to identify recipient central brain regions of OPN3-RGCs.

#### RGR and RRH

Photoisomerases are opsins whose primary role is to convert retinoids from *trans* into *cis* as fresh fuel for light-sensing opsins. In the mammalian eye, *Rgr* and *Rrh* (also called “peropsin”) are expressed exclusively in cells of the RPE and Müller glia cells [14, 84]. The deletion of *Rgr* in mice leads to an aberrant accumulation of *cis* retinaldehyde isoforms (higher levels of *9-cis* and *13-cis*, instead of the preferred *11-cis* isomer) [85], and in humans, mutations in *Rgr* are associated with retinitis pigmentosa [86]. No G-protein signaling has been reported for *Rgr*, and the necessary intracellular amino acid motifs for G-alpha binding and activation are not conserved compared to other opsins [31]. Combined, these results suggest that RGR acts as a photoisomerase. While *Rrh* is similarly expressed in the RPE, neither direct evidence of photoisomerase or G-protein activity has been detected in the wild-type protein, and a role as a shuttle for *trans* retinoids has been suggested [87, 88]. Because these opsins have not been found to contribute significantly to extraocular vision in mammals, birds, or fish, further emphasis will not be given to them here. However notably, the arachnid *Rrh* acts as a G-protein activating dark-sensor, which is inactivated by light [89].

### Circadian rhythms

The majority of Earth’s species of plants, fungi, bacteria, and animals contain endogenous circadian clocks. These circadian clocks approximate the rotation of the earth, and they allow for organisms to anticipate the repetitive 24-h changes in light, temperature, and humidity in their environment. It has not always been appreciated that these behavioral rhythms were intrinsic rather than driven by the environment. In 1729, the French scientist Jean-Jacques de Mairan observed that the *Mimosa pudica* plant exhibited circadian leaf movements even when shielded from rhythmic solar cues [90]. However, he and his contemporaries suspected that the plants had a way of detecting the sun even when isolated from its light. In 1971, Ronald Konopka and Seymour Benzer published the results of a mutagenesis screen on *Drosophila melanogaster* fruit flies in which they screened for circadian clock mutants [91]. The three mutant fly lines they characterized had circadian clocks that ran faster than 24 h (about 19 h), slower than 24 h (about 28 h), or showed no rhythmic behavior at all. This work provided definitive proof that (1) the circadian rhythms the animals were displaying were intrinsic and not driven by the sun or some unseen time-cue, and (2) it showed that the circadian clock was governed by a heritable molecular mechanism. The three mutations were later localized on the same gene, *period* [92, 93]. A similar mutagenesis screen in the 1990’s by Joseph Takahashi showed that single, heritable gene mutations also controlled the period length and integrity of circadian activity in mammals [94–96].

**Fig. 2:**
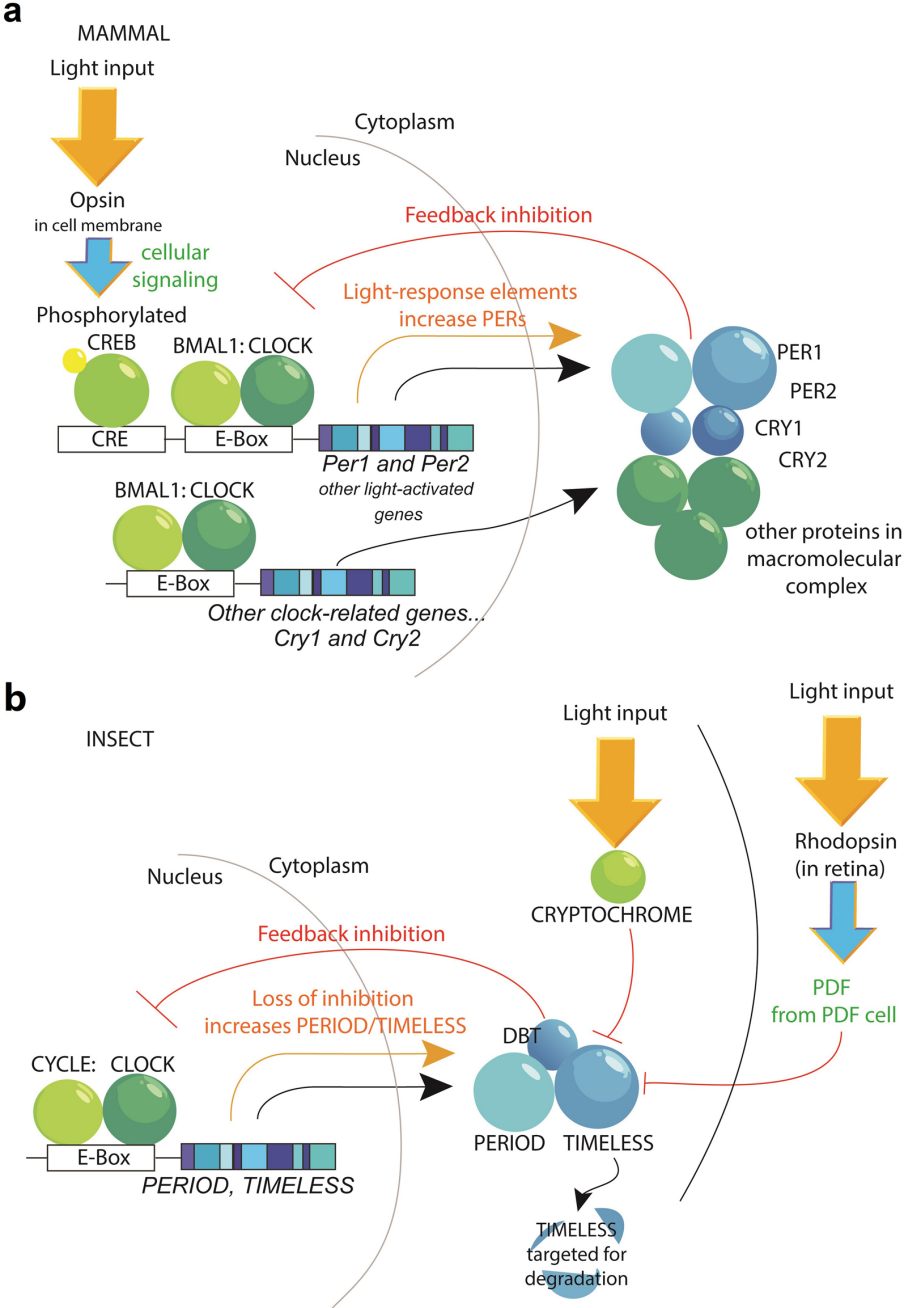
Light input into the molecular circadian clocks of mammals and insects. For clarity, these simplified models focus on light input and omit other clock-related factors. **a** In the mammalian circadian clock, the CLOCK:BMAL1 transcription complex initiates transcription of clock-controlled genes such as *Per1*, *Per2*, *Cry1*, and *Cry2*. The respective proteins of these genes form a macromolecular complex in the cytoplasm which translocate into the nucleus, where it represses further CLOCK:BMAL1-mediated transcription. Light input (orange arrow) activates an opsin in a retinal ganglion cell or a neighboring opsin-expressing cell type. When the light-related signal is received by the clock-expressing cell (such as the case of an SCN neuron being activated by glutamate or PACAP from the retina), this causes a phosphorylation of CREB which binds to CREB response elements (CRE) in *Per1* and *Per2* genes. The direct increase in transcription of these genes is associated with shifts in the circadian clock. **b** Similarly, in insects, CYCLE:CLOCK transcriptional machinery initiates the transcription of clock-related genes such as *period* and *timeless*. The PERIOD and TIMELESS proteins form a complex, including DOUBLETIME (DBT), which inhibits further CYCLE:CLOCK-mediated transcription. In contrast to the mammalian system, light input causes the degradation of TIMELESS, which destabilizes the repressor complex, and relieves transcription inhibition of CYCLE:CLOCK. Degradation of TIMELESS is either initiated by direct light activation of CRYPTOCHROME, or indirectly through rhodopsin activation in the retina, which activates pigment dispersing factor (PDF) cells in the brain, causing degradation of TIMELESS in clock neurons

Over the centuries since de Mairan’s mimosa plants swayed in complete darkness, the persistence of near-24-h rhythms were demonstrated in a myriad of organisms as diverse as unicellular dinoflagellates [97] to human beings [98]. The rare examples of arrhythmic animals in deep caves, extreme depths of the ocean, or near the poles of Earth are often found to have lost the circadian mechanism through mutation (suggesting their ancestors once had functional clocks) or still have functioning molecular clocks that uncouple from behavior [99–102]. It is now well accepted that having a circadian clock is expected in almost every creature investigated and discovering a lack of rhythmic features is an anomaly. The molecular mechanisms of rhythms in bacteria [103], plants [104, 105], fungi [106–108], insects [92, 93, 109], and mammals [94–96, 110, 111] have been extensively studied and show remarkable similarity in the presence of a negative feedback model between transcriptional and post-translational elements [112, 113]. Briefly, transcription factor proteins produce their own inhibitory proteins to produce a cycle with oscillating positive and negative transcriptional states (Fig. 2a) [113]. Post-translational modifications to proteins, chromatin modifications, and associations between large protein complexes allow the molecular cycle to be tailored to near-24-h precision [111, 114, 115]. Intriguingly, much simpler molecular circadian rhythms have also been observed in cyanobacteria and in mammalian red blood cells that do not require a transcriptional component [116–118]. While the transcriptional-repressor model is nearly ubiquitous among living things, researchers should remain open to alternative parallel mechanisms.

At about the same time that Konopka and Benzer were screening mutant *Drosophila* strains, anatomical experiments were underway to determine the brain regions responsible for behavioral circadian rhythms in multiple species. A couple of publications in 1972 revealed that the SCN of the mammalian anterior hypothalamus was necessary for behavioral circadian rhythms in rodents [119, 120]. In birds, the anatomy is slightly more complex, with regulation of behavioral circadian rhythms involving the pineal gland, the SCN, and additional hypothalamic regions [121–124]. In addition to the astounding revelation that the clock’s molecular mechanism is similar among all animals, the majority of central anatomical clocks in mammals operate, at least in part, using diffusible paracrine factors. In insects [125], birds [123], and mammals [126], transplantation of brain regions associated with behavioral clock regulation imparted the phase and period of the donor onto a recipient host. Even when the transplanted SCN is encapsulated in a semi-permeable membrane, the diffusible signals are sufficient to restore some of the circadian rhythmic patterns of locomotor activity in hamsters [127].

### Photoentrainment of circadian rhythms

The free-running period of an individual cell within a tissue or individual animal within a population may be quite precise, but differences between individual cells are distributed over a range of periods. Thus, over many cycles the phases of individual free-running clocks would become scattered. In order for circadian clocks to be useful at a population level or as an ecological strategy, they must be synchronized to a steady cue. The vast majority of organisms synchronize their circadian rhythms to light in their environment (“photoentrainment”) using photoreceptors that couple the near-24-h molecular clock to the exactly 24-h light cycle. This review will focus on circadian photoentrainment in animals, although there has been extensive work exploring photoentrainment in unicellular organisms [128], plants [129], and fungi [130]. Both circadian entrainment to a 24-h cycle and photoperiodism (the anticipation of varying seasonal daylength) will be considered together because their mechanisms are related, yet distinct. In all cases that have been uncovered, photoreception for circadian photoentrainment and photoperiodism involve extra-visual pathways.

#### Insects

The central behavioral circadian clock network in insects is distributed throughout the brain in the form of about six small clusters of neurons wired together in a clock circuit [131]. Within a subset of these cells, the protein CRYPTOCHROME is expressed and acts as a photoreceptor [132–135]. CRYPTOCHROME is a flavoprotein photoreceptor containing the short-wavelength light-sensitive flavin adenine dinucleotide (FAD) which acts as an allosteric switch [136]. When activated by light, conformational changes in the structure of CRYPTOCHROME cause it to bind to TIMELESS and target the protein complex for degradation (Fig. 2b) [137]. TIMELESS is a critical state-variable protein in the inhibitory limb of the fly’s circadian clock, so its degradation causes a resetting of the overall clock phase. CRYPTOCHROME requires light of relatively high intensity for activation; however, the entrainment of fly behavior also occurs at lower light intensities. The opsin Rhodopsin 7 (Rh7) functions as an additional circadian violet-light-detector in clock neurons at lower light intensities [138]. Light can induce these mechanisms directly within the clock neurons, leading to a shift of the behavioral clock outside of the visual system [134]. However, there is also input from the fruit fly’s eyes on behavioral rhythms. Here, “eyes” includes the large compound eyes, eyelets, and ocelli which all express opsins and contribute to behavioral photoentrainment [139]. The opsins expressed in these structures consist of rhodopsins 1–6 (Rh1*–*Rh6) which mediate vision as well as circadian responses [140–142]. Only when the brain photoreceptors and the eyes have been disabled or eliminated do the flies’ clocks become behaviorally insensitive to light [143].

Photoperiodism was also found to be regulated by non-ocular photoreceptors in insects by experiments by Lees in the late 1950s and 1960s. Lees found that photoperiod changes stimulated the egg laying state of aphids much more strongly when the light was administered to the central head than light administered to the eyes [144]. Finally, some insects, such as the monarch butterfly, integrate magnetic signals and non-visual light cues using two paralogs of CRYPTOCHROME to adjust magnetoreception for the feat of long-distance migration [145–147]. The elegant mechanism merges signals from circadian photoentrainment, photoperiodism, and Earth’s magnetism to achieve a global positioning system within the butterfly’s brain.

#### Birds and reptiles

Similar to *Drosophila*, birds contain redundant or parallel photoreceptive elements to achieve circadian and photoperiodic behavior. In 1935, Jacques Benoit observed that male ducks would enter the seasonal breeding state of enlarged gonads even in the absence of eyes, or when light was delivered directly into the brain’s third ventricle [148]. Extensive work was performed by Michael Menaker and colleagues in the 1960s and 1970s in the sparrow delineating the photoreceptive structures for circadian and photoperiod measurement. Sparrows with eyes surgically removed maintained behavioral photoentrainment to a 24-h light:dark cycle nearly identically to sighted birds [149]. Similar to Benoit’s ducks, male eyeless sparrows also entered their reproductive state when given long photoperiods characteristic of summer [150]. It was only when Menaker and his colleagues obscured the top of the skull with dark ink that the blinded sparrows stopped exhibiting circadian photoentrainment, and even with eyes intact, obscuration of the top of the skull blocked photoperiod measurement [151]. These results led to the discovery of the pineal gland as a seasonal light-sensing organ in sparrows. The avian and reptilian pineal glands and parietal eyes (reptiles only) express non-visual opsins, such as pinopsin, parapinopsin, and parietopsin, which are not found in mammals [152–154]. The opsins in these structures contribute to the extra-visual non-retinal photoreception separately from pattern image forming vision in a manner that is common among the vertebrates.

Birds have yet another photosensitive brain region in addition to the eyes and pineal: the paraventricular organ (PVO) of the hypothalamus [155, 156]. Male quails respond to photoperiod changes even with both eyes and pineal glands removed [62, 157, 158]. The laboratory of Takashi Yoshimura identified neurons in the quail hypothalamus that have cilia that reach into the third ventricle and express the non-visual opsin *Opn5* [62]. These *Opn5*-expressing neurons exhibit intrinsic light responses of action potential firing even under pharmacologic blockade of upstream neurons, demonstrating their true status as deep-brain photoreceptors [159]. The presence of OPN5 and other opsins in similar regions in fish and mice suggests the potential of a shared ancestral lineage of hypothalamic photoreceptors [67, 68, 160].

Similar to birds, reptiles do not require their eyes for circadian photoentrainment or photoperiod measurement [161]. While the eyes and the pineal gland both influence circadian photoentrainment, both can be removed and at least some species of lizard maintain behavioral photoentrainment [161, 162]. In fact, removal of the eyes alone reveals stronger circadian light responses than sighted animals of the species *Sceloporus occidentalis* [163]. Because covering the head with black paint can block light responses and injecting antisense RNA against opsins into the third ventricle also blocks photoentrainment, it is likely that a hypothalamic photoreceptive system exists in lizards as it does in birds and fish [161, 164].

#### Fish

Fish use a combination of opsins [165] and a photoreceptive *Cryptochrome* [166] for circadian photoentrainment*.* Fish have a particularly decentralized circadian system in which all organs express robust internal rhythms capable of oscillating in their own phases without much central control [165, 167]. While a hypothalamic SCN in teleost fish exists and expresses clock genes, its role in behavioral rhythms is not as clear as in mammals [165, 168]. The pineal gland plays a more central role in the regulation of behavioral rhythms in fish as compared to mammals [169]. However, other sleep-promoting centers of the teleost brain are remarkably similar between fish and mammals [170]. In both, serotonin production by the raphe nucleus, prokineticin 2, and histamine are involved in the regulation of sleep and sleep drive [171–173]. Most importantly, the release of melatonin from the pineal gland during the dark phase of a light:dark cycle is central to the rhythmic nature of sleep in fish, birds, and mammals [174, 175]. Much of our understanding of fish circadian rhythms comes from the study of zebrafish and medaka. As more species of fish are studied, more interesting details and variations will likely come to light.

#### Mammals

The eyes are essential for all behavioral circadian photoentrainment in all mammals that have been tested. Eyeless ground squirrels, mice, and hamsters show no evidence of behavioral circadian light-responses even when exposed to direct sunlight [176, 177]. However, the absence of non-ocular photoentrainment is not evidence for vision-based photoentrainment. Many blind people, with no conscious perception of light, experience no difficulty maintaining normal patterns of sleep: activity rhythms, while others have difficulty maintaining 24-h schedules [178, 179]. Charles Czeisler and colleagues showed that for some people with no visual light perception, the nocturnal suppression of melatonin by light was preserved [180]. Blindfolding the light-responding patients eliminated the photo-suppression of melatonin [180]. Clearly, the eyes were necessary for behavioral entrainment, but vision was not.

Identification by Clyde Keeler in 1923 of a naturally occurring mutation in mice which causes severe outer retinal degeneration set off decades of research on non-visual circadian responses in mice [181, 182]. These mice lacked rods and cones, and showed no evidence of vision, yet their pupillary light reflex was similar to sighted mice (recall Brown-Séquard’s eel pupil) [183]. A similar strain of sightless mice with retinal degeneration was found to entrain almost normally to 24-h light:dark cycles by Ebihara and Tsuji in 1980, prompting them to predict “that at least two different kinds of photoreceptor including rods and cones participate in the photic entrainment of the circadian activity rhythms” [184]. Using various targeted mutations of mouse visual pathways, the lab of Russell Foster demonstrated conclusively that while the eyes were necessary for behavioral photoentrainment in mice, vision was not [185]. The burgeoning field of non-visual circadian photoreception was ignited into a flurry of ground-breaking discoveries with the identification of OPN4 (melanopsin) in RGCs in the early 2000s [37, 38, 186, 187]. A couple of papers in *Science* in 2002 demonstrated that these OPN4-containing ganglion cells make a single synaptic connection in to the SCN, and that they are intrinsically light sensitive [186, 187]. Multiple laboratories then genetically deleted melanopsin and showed that, similar to either the eyes or the pineal entraining clocks in sparrows, either melanopsin or rod/cone pathways were sufficient for behavioral photoentrainment in mice [188, 189]. An elegant genetic strategy was then employed by the laboratories of Samer Hattar and Satchidananda Panda to ablate just the OPN4-containing cells themselves. This work showed that the subset of rods and cones which contribute to circadian alignment send their signals (alongside OPN4 signaling itself) through the OPN4-ganglion cells to relay their signals to the SCN [190, 191]. Thus, even in sighted mice, the absence of OPN4-expressing RGCs abolished behavioral circadian photoentrainment. In a parallel to Keeler’s 1927 observation that the pupillary light reflex persists in retinally degenerated mice [183], OPN4-positive ganglion cells also project to the OPN, where they play a critical role in regulating pupillary constriction [186, 188, 192, 193].

Although not necessary for behavioral photoentrainment, OPN5 plays a role in mammalian behavioral photoentrainment as well. Two independent studies found behavioral circadian deficiencies in mice lacking *Opn5* compared to wild-type mice [66, 194]. The *Opn5*-null mice required extra days to resynchronize behavioral rhythms to a shifting light:dark cycle in a jet-lag protocol, and they showed an inability to synchronize to dim violet light levels sufficient for wild-type entrainment [194]. Interestingly, OPN5-expressing RGCs do not directly innervate the SCN to a large degree, suggesting a more circuitous route by which OPN5 may influence circadian behavior, another brain region contributing to behavioral photoentrainment, or the influence of peripheral clocks on behavioral photoentrainment [67, 68, 78].

In summary, it is remarkable that in all animals studied redundant or parallel light input mechanisms exist for photoentrainment and photoperiodism. Synchrony to environmental oscillations represents such a strategic advantage that evolution has converged on and conserved multiple photoreceptors in all creatures. In studies on fish, insects, birds, and mammals, a few well-studied species often represent the vast diversity of each group, so many unique adaptations and exceptions likely remain undiscovered [195, 196].

### Extra-behavioral photoentrainment

In addition to the overt circadian rhythmicity of animal behavior, if one can peer into the individual cells within an animal, the majority of cells and tissues have molecular clocks ticking with a precise, near-24-h rhythm. At the level of tissue, this was demonstrated by Erwin Bünning in 1958 by observing that an isolated segment of hamster intestine maintained a circadian rhythm of contraction even when explanted from the body [197]. Once the field of molecular biology caught up to the field of physiology, it was shown that most cells also exhibited circadian oscillations [198–202]. The idea of the SCN being the mammalian central time driver gave way to the idea that it was more of a central synchronizer [203]. However, there is not a single golden signal from the SCN which synchronizes all clocks throughout the body. Body temperature [204, 205], glucocorticoids [206, 207], melatonin [208], and autonomic innervation [209] have all been shown to have influence on the phasing of individual tissue clocks. Intriguingly, it became clear that peripheral tissue clocks could become unmoored from SCN control and adopt new SCN-independent phases of oscillation. The laboratories of Ueli Schibler and Michael Menaker showed that when food availability and a light:dark cycle were presented in opposition, the phase of the liver molecular clocks inverted their relationship to the SCN clock [210, 211]. When the circadian clock of the SCN was genetically disabled, transcriptomic analysis of the liver revealed that SCN-related systemic cues and feeding-cycle-driven cues comprised parallel inputs to the liver’s circadian clock with certain rhythmic elements responding to only one or the other cue within the same cells [212].

If light can photoentrain the central brain clock, can it also act as a direct time cue for peripheral circadian clocks? In fruit flies and zebrafish, there is an extensive photoentrainment of most peripheral clocks. Using a GFP reporter linked to the circadian gene *period* (the same gene mutated in Konopka and Benzer’s 1971 screen), the lab of Steve Kay found that the circadian clocks of most fly body parts, such as the proboscis, antenna, leg, and wing, were directly photoentrainable to light cycles as explants [213]. When multiple potential synchronizing modalities are present in conflicting phases, light vs*.* temperature, for example, *Drosophila* peripheral clocks utilize light as their primary synchronizing cue [214]. However, this peripheral photoentrainment is not due to non-visual opsins: the peripheral fly clocks were photoentrained by the CRYPTOCHROME degradation mechanism [134, 214, 215]. In contrast, peripheral organs in fish do use opsins for photoentrainment, at least in part. Remarkably, tissues deep within the zebrafish body, such as the heart and kidney can be directly photoentrained [216]. Multiple opsins are expressed throughout the fish body including TMT-opsin, Opn3, Opn4, Opn5, and exorhodopsin [25, 29, 74, 217, 218]. The exact roles of these extraocular opsins are still being uncovered, and some may be primarily for local regulation of features like skin color and not circadian in nature [219]. A tantalizing study by Foulkes’ group analyzed deep cavefish that lost behavioral and peripheral circadian rhythms [99]. Mutations were identified in the OPN4 and TMT opsins in cavefish compared to zebrafish, and when sequences were replaced with wild-type zebrafish sequence, the circadian photoentrainability of the cavefish cells was restored [99]. Furthermore, some of the light-sensitive DNA repair photolyases, which repair UV-damaged cyclobutane pyrimidine dimers (CPD), have been mutated to lose photoreceptive qualities in cavefish, but the CPD photolyases retain their light-independent repair capabilities [220]. While not necessarily circadian in nature, cells of the pituitary gland in medaka fish also use OPN5 to directly sense short-wavelength light to release melanocyte stimulating hormone (MSH) to influence body coloration [221].

Birds and mammals do not show the same degree of direct photoentrainment of peripheral organs as flies and fish, but significant examples have been demonstrated. In birds, pineal glands and pinealocytes can be cultured ex vivo, and their production of melatonin remains photoentrainable [222]. Similarly, the melatonin production of hamster retinas remains directly synchronized by light in culture [199]. Using murine retinas, our laboratory found that the rhythms of the circadian clock reporter *Period2:Luciferase* was also synchronized directly by light ex vivo [223]. Remarkably, we found that rods, cones, and *Opn4* were not necessary for the photoentrainment of mouse retinas, rather mammalian *Opn5* was required [66]. Rhodopsin has also been implicated in the mechanism of mammalian retinal photoentrainment [224]. Even in vivo, mice without *Opn4*, rods, and cones showed no behavioral photoentrainment, but their retina clocks maintained local molecular photoentrainment [223]. We then analyzed other tissues which had been shown to express OPN5, like skin [64]. The circadian clocks of mouse skin also photoentrained to light:dark cycles ex vivo, similar to organs from zebrafish and drosophila [65]. Unlike zebrafish, no photoentrainment was observed in tissues from deep within the mouse’s body [65, 66, 68]. Mouse skin in culture required an exogenous source of *cis* retinaldehyde similar to the chromatophore expansion in cultured frog melanocytes [35]. When mice without *Opn4*, rods, and cones were allowed to free-run through a violet-light containing light:dark cycle, murine skin, particularly heavily exposed skin such as the ear’s pinna and vibrissal pad, was directly photoentrained, independent of the phase of the animals’ behavioral rhythm [65].

We also observed a direct *Opn5*-dependent photoentrainment of the circadian clocks within explanted murine corneas [69]. However, when the phases of clocks in corneas in free-running blind mice in vivo were analyzed, the corneas tracked the body’s clocks, not the light-cycle [69, 225]. In corroboration with work by Tosini and Menaker, it was observed that melatonin and glucocorticoids have a large influence on the phase of the corneas’ clocks in vivo [208, 226, 227]. The expression of *Opn5* is barely detectable in a healthy cornea and is induced to a much higher level after wounding [70]. This facultative expression of OPN5 induces a violet-light sensitivity that accelerates healing of the corneal surface [70]. Once the cornea heals, the *Opn5* expression returns to low, baseline levelsThis temporary, violet-light sensitive healing mechanism occurs in both rodent and primate corneas [70]. It is still unclear whether the temporary light-sensitivity of the circadian clock in wounded corneas influences the healing rate, or if it is non-clock-related photo-sensitive elements. It will be interesting to determine if other, non-mammalian species also express facultative extra-visual opsins in response to wounding.

*Opn3* is not required for the photoentrainment of peripheral circadian clocks in retinas or corneas in mammals [66]. However, it may play a light-independent role in peripheral clocks. The amplitude (the peak-to-trough ratio) of the oscillations of the circadian *Per2:Luciferase* reporter [203] were reduced in *Opn3*^*−/−*^ retinas and white adipose tissue [31, 66]. Similarly, the diurnal differences in cAMP levels in white adipose tissue were also blunted in the absence of *Opn3* [26]. Whether this light-independent role for OPN3 is related to its pairing with other receptors, such as the MCR1 and MCR4, remains unclear [27, 28].

## Conclusions

Being able to predict the daily cycles of light and dark must confer survival advantage to animals able to do so. Undoubtedly, this selective advantage has been the evolutionary pressure that has resulted in circadian clock mechanisms being a nearly ubiquitous feature of animal life on earth. Animals of multiple phyla including insects and chordates have adopted similar molecular mechanisms, using conserved genes like *period*, to establish a free-running clock. In most animals, many tissues and cell types are capable of maintaining free-running circadian rhythms when cultured in isolation. Animals have evolved mechanisms to maintain internal synchrony of these clocks, which typically involves the evolution of brain regions (such as the SCN in mammals and the pineal in birds and lizards) capable of generating hormonal or other signals to ensure clock synchrony throughout the body. To be useful, clocks must also maintain synchrony with the solar light–dark cycle. Remarkably, in all animals from insects to humans, multiple redundant mechanisms appear to maintain entrainment of these circadian clocks to the 24-h light–dark cycle. In *Drosophila*, for example, the visual system is sufficient to entrain behavioral rhythmicity but is not necessary. Similarly, *cryptochrome* function is also sufficient but not necessary for behavioral entrainment. Only when both are lost do animals become behaviorally arrhythmic. Further, peripheral tissues in *Drosophila* are directly photoentrainable [213], and require the non-visual photopigment *cryptochrome* for this function [228, 229]. The same hierarchy appears to function in mammals, with both visual system photopigments and inner retinal melanopsin each sufficient but not necessary for behavioral circadian entrainment, and numerous peripheral tissues including cornea and skin directly photoentrainable via a non-visual photopigment (in this case OPN5). The subtleties of function that have preserved these multiple, partially redundant photoentrainment systems are not yet clear.

It is also clear that each of these pathways may subserve multiple functions besides circadian entrainment. The visual photopigments’ primary role, of course, is to subserve form vision. OPN4, in addition to mediating circadian entrainment, also mediates the pupillary light response, direct stimulation of sleep and wakefulness, and development of retinal vasculature among other functions. OPN5 appears to play a significant role in conferring light-sensitive acceleration of wound healing in the injured cornea. Ongoing research will undoubtedly reveal additional critical roles for these non-visual photoreceptors in diverse physiological systems throughout the body.

## Data Availability

Not applicable.
